# Retinal nitric oxide and malonyldialdehyde levels following photodynamic therapy

**DOI:** 10.4103/0301-4738.73705

**Published:** 2011

**Authors:** Peykan Türkçüoğlu, Cem Öztürkmen, Nevin İlhan, Julide Kurt, Orhan Aydemir, Ülkü Çeliker, Mohamed A İbrahim, Aymen Rashid

**Affiliations:** Department of Ophthalmology, Firat University School of Medicine, Elaziğ, Turkey; 1 Department of Ophthalmology, Goznur Eye Hospital, Gaziantep, Turkey; 2 Department of Biochemistry, Firat University School of Medicine, Elaziğ, Turkey; 3 Department of Ophthalmology, Wilmer Eye Institute, Johns Hopkins University School of Medicine, Baltimore, Maryland

**Keywords:** Malonyldialdehyde, nitric oxide, photodynamic therapy, retina

## Abstract

**Background:**

To determine the retinal nitric oxide (NO) and malonyldialdehyde (MDA) levels following photodynamic therapy (PDT).

**Materials and Methods:**

Seven Dutch-belted rabbits received dextrose, while seven others received 2 mg/kg verteporfin infusion over a period of 15 minutes in a dim-lit room. Irradiation to a 1.5 mm diameter intact chorioretinal area in the right eye of verteporfin-infused rabbits, was started 5 minutes after the end of infusion. Three groups were control (dextrose infusion), infusion with verteporfin (left eyes were not irradiated), and irradiation after verteporfin injection (right eyes were irradiated). On the fifth day of the experiment, the eyes were enucleated. The retinas were subsequently frozen and homogenized. Nitrite, a stable end-product of NO and MDA, was measured using the spectrophotometer. Protein concentrations were measured by the Lowry method. Tissue NO and MDA levels were expressed as μmol/gprt and nmol/mgprt, respectively.

**Results:**

The mean retinal NO and MDA levels of the control, infusion, and irradiation groups were 24.67 ± 6.66, 0.11 ± 0.02; 45.90 ± 15.52, 0.21 ± 0.09; and 84.43 ± 14.96 μmol/gprt, 0.58 ± 0.14 nmol/mgprt, respectively. The mean retinal NO levels were significantly elevated in the infusion and irradiation groups compared with the control group (*P*:0.004; *P*:0.001). The mean retinal MDA levels were significantly elevated in the infusion and irradiation groups compared to the control one (*P*:0.026; *P*:0.001). Also the mean retinal NO and MDA levels in the irradiation group were found to be significantly higher than the infusion group (*P*:0.018; *P*:0.018).

**Conclusion:**

Not only PDT, but also verteporfin infusion alone resulted in NO and MDA level increments in the retina, which might be toxic.

Choroidal neovascularization (CNV), a major clinical complication of ocular angiogenesis, is an important cause of vision loss that affects a large number of people worldwide, especially in the elderly. It refers to the formation of new blood vessels that arise from choriocapillaris through the Bruch’s membrane and breaks into the subretinal space.

Photodynamic therapy (PDT) is one of the procedures available for the treatment of patients with CNV. It is based on systemically applied verteporfin, which upon activation with agent-specific non-thermal light, produces singlet oxygen and / or other reactive intermediates.[1,2]

Nitric oxide (NO) is generated by the oxidation of arginine by the enzyme nitric oxide synthase (NOS). Although small amounts of NO mediate physiological functions, excess NO can mediate tissue injury. Previous studies demonstrated that NO is toxic to photoreceptors.[3–5]

Malonyldialdehyde (MDA) levels indicate the amount of cellular damage secondary to lipid peroxidation. As the direct measurement of liberated free radical species is limited by their instability, the level of MDA, the stable product of oxidative degradation of polyunsaturated fatty acids, has been widely adopted as a measure of free radical formation.[6]

The purpose of this study is to determine the retinal NO and MDA levels in sensitizing light-exposed and unexposed eyes following PDT.

## Materials and Methods

Fourteen pigmented Dutch-belted rabbits weighing between 2500 and 3000 g were treated in accordance with the Association for Research in Vision and Ophthalmology, in the Experimental Research Center, on the consent of the local ethics committee of our University. Intramuscular ketamine HCl (50 mg/kg) and xylazine HCl (5 mg/kg) were used as anesthesia for all the rabbits. Half of the rabbits received 6 ml dextrose infusion and the other half received 2 mg/kg verteporfin (Visudyne^®^; Novartis, Switzerland) infusion for 15 minutes in a dim-light room.[7] Irradiation was started 5 minutes after the end of infusion to the right eyes, using a conventional diode laser / slit lamp setting (Visulas; Zeiss, Germany), to the verteporfin-infused rabbits. A single laser spot, 1.5 mm in diameter, was applied to an intact chorioretinal area at a dose of total exposure of 50 J/cm^2^ over 83 seconds. The groups were divided as:

Control Group: The rabbits that were given dextrose infusion (The average of both eye measurements was used in the statistical analysis).

Infusion Group: The left eyes that were not irradiated after the infusion of verteporfin.

Irradiation Group: The right eyes that were irradiated after the infusion of verteporfin.

Rabbits were kept in a dark room for 48 hours. On the fifth day of the experiment, the rabbits were sacrificed by intracardiac thiopental sodium (50 mg/kg). Both eyes of all the rabbits were enucleated rapidly. The enucleated eyes were dissected coronally through the pars plana. After removing the vitreous, the retinas were gently peeled off and cut from the optic disc with fine forceps and scissors under an operating microscope and washed with phosphate buffered saline (PBS) (pH 7.4 molar 0.2). The retinas were frozen and homogenized in PBS at a dilution of 1/20. The supernatants were collected by centrifugation at 150 g for 10 min, and stored at −80°C until the assay.

Nitrite, a stable end-product of NO, was measured by using the spectrophotometric Greiss reaction.[8] One milliliter experimental samples of deproteinized (NaOH-ZnSO4 deproteinization method was used) supernatant were reacted with 500 μl N-naphthylethylenediamine, 1% sulfanilamide for 45 minutes at room temperature and analyzed by spectrophotometry at 545 nm. The concentrations were determined by comparison with sodium nitrite. The tissue NO levels were expressed as μmol/g protein.

Malonyldialdehyde was measured spectrophotometrically as described by Ohkawa.[9] pH was adjusted to 3.4 with the aid of a pH meter and the homogenate was incubated at 95°C for one hour after addition of 1.5 ml %0.8 thiobarbituric acid (TBA). The pink-colored complex that formed was measured spectrophotometrically at 532nm. 1,1,3,3, tetraetoxypropane was used as a standard. Tissue MDA levels were expressed as nmol/mg protein. Protein concentrations were measured by the Lowry Method.[10]

Statistical analyses were carried out by employing the Statistical Package for Social Sciences soft-ware 13.0 for Windows package software (SPSS, Inc., Chicago, IL). The Mann-Whitney U and Wilcoxon signed-ranked test were used for independent variables and dependent variables, respectively. *p* values less than 0.05 were considered as statistically significant.

## Results

**Figure 1:**
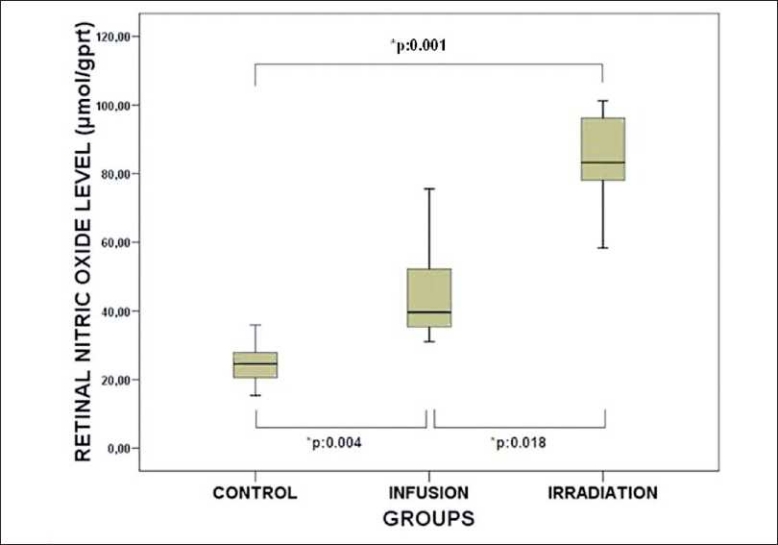
Retinal NO levels from control, infusion, and irradiation groups. Significant differences are demonstrated with * p. The black lines in the box plot diagram show the median values of the groups. (Mann-Whitney U test was used in the statistical comparison of the control and infusion, and control and irradiation groups. Wilcoxon signed-ranked test was used in statistical comparison of infusion and irradiation groups)

**Figure 2:**
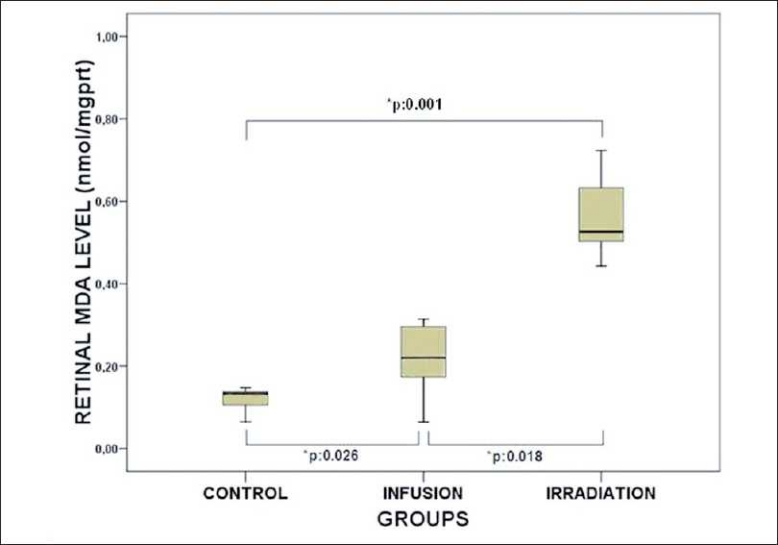
Retinal NO levels from control, infusion, and irradiation groups. Significant differences are demonstrated with * p. The black lines in the box plot diagram show the median values of the groups. (Mann-Whitney U test was used in statistical comparison of control and infusion; control and irradiation groups. Wilcoxon signed-ranked test was used in the statistical comparison of the infusion and irradiation groups)

The mean retinal NO levels of the control, infusion, and irradiation groups were 24.67 ± 6.66, 45.90 ± 15.52, and 84.43 ± 14.96 μmol/gprt, respectively. The mean retinal MDA levels of the control, infusion, irradiation, groups were 0.11 ± 0.02, 0.21 ± 0.09, and 0.58 ± 0.14, nmol/mgprt, respectively. The mean retinal NO levels were significantly elevated in the infusion and irradiation groups compared to the control group (Mann-Whitney U test: p:0.004; p:0.001) [Fig. 1]. Also the mean retinal NO level in the irradiation group was found to be significantly higher than that in the infusion group (Wilcoxon signed-ranked test: p:0.018) [Fig. 1]. The mean retinal MDA levels were significantly elevated in the infusion and irradiation groups compared to those in the control group (Mann-Whitney U test: *p*:0.026; *p*:0.001) [Fig. 2]. Also the mean retinal MDA level in the irradiation group was found to be significantly higher than that in the infusion group (Wilcoxon signed-ranked test: p:0.018) [Fig. 2].

## Discussion

Photodynamic therapy with verteporfin has been demonstrated to be an effective treatment for CNV. The superiority of PDT is that it preserves retinal tissue as compared to the conventional laser. However, in animal models, it has been seen that limited damage is still inflicted on the retina.[11–13] Most of the patients with CNV need repeated treatments, so the cumulative damage becomes an important concern.

NO is not only an important regulator of cell functions, but also has a role in cellular injury and death, by reacting with the superoxide to form peroxynitrite. Unregulated NO production causes apoptosis or cell death.[14] In human embryonic kidney cell models, it has been demonstrated that NO is responsible for cell susceptibility with delta-aminolevulinic acid-induced PDT.[15] In different studies, by using tumor tissue or cell lines, it has been seen that different photosensitizing chemicals mediate photo-induced cell death via NO.[16,17] In a model of CNV, NO increments have been documented following PDT and it is found to be related to photoreceptor damage.[18] In our study, we have demonstrated that not only verteporfin activation by irradiation, but also its infusion without irradiation increases NO level in the retina.

Photoactivation of verteporfin is known to generate reactive oxygen species (ROS), therefore, there is oxidative stress. Oxidative stress causes damage to cellular macromolecules, such as, nucleic acids, proteins, and lipids. Among these, peroxidation of lipids leads to facile propagation of free radicals. MDA is one of the products of lipid peroxidation that is stable.[6] Following PDT with different photosensitizing chemicals, MDA level increments were documented in cell lines and tumor models.[19,20] However, the effect of PDT on the MDA level in an eye model has not been studied yet. In our study, we demonstrated that MDA increases both after infusion and irradiation. The mean MDA level in the irradiation group was found to be significantly higher than the infusion group. This indicates that the photoactivation of the verteporfin molecule results in a free radical burst as expected.

Nitric oxide and MDA levels in the retina were found to be elevated in the infusion group compared to the control group. We speculated that a fraction of circulating verteporfin in the choroid and retina was activated without irradiation with a non-thermal laser, for example in a dim-room light, which was the standard room condition during the 15-minute verteporfin infusion. This was the conflict in relation to the working mechanism of verteporfin. However, it was the only possible manner in which to explain our result.

Tissues are normally protected from oxidative damage by the presence of enzymes, such as, superoxide dismutase and catalase. The presence of these enzymes has been seen in the retina.[21] However, if the amount of free radicals exceeds the defensive capacity of the cells, this leads to death. In our study, for the first time, we have demonstrated the elevation of NO, which may also increase peroxynitrite via the reaction of the superoxide with MDA, which is the stable product of oxidative degradation of polyunsaturated fatty acids following verteporfin infusion alone. In the clinical setting, this infusion-related free radical burst may result in tissue injury in the fellow eye of the patient in unilateral CNV cases. In order to give an exact clinical suggestion, which is also the shortcoming of our study, further studies, including apoptosis evaluation and / or electrophysiological measurement from the retina must be conducted, to investigate whether or not the CNV-free fellow eyes can combat this free radical liberation.

In conclusion, PDT as well as verteporfin infusion alone, resulted in NO and MDA level increment in the retina.

## References

[1] van den Bergh H (2001). Photodynamic therapy of age-related macular degeneration: History and principles. Semin Ophthalmol.

[2] Sharman WM, Allen CM, van Lier JE (1999). Photodynamic therapeutics: Basic principles and clinical applications. Drug Discov Today.

[3] Ju WK, Chung IW, Kim KY, Gwon JS, Lee MY, Oh SJ (2001). Sodium nitroprusside selectively induces apoptotic cell death in the outer retina of the rat. Neuroreport.

[4] Donovan M, Carmody RJ, Cotter TG (2001). Light-induced photoreceptor apoptosis *in vivo* requires neuronal nitric-oxide synthase and guanylate cyclase activity and is caspase-3-independent. J Biol Chem.

[5] Osborne NN, Wood JP (2004). Metipranolol blunts nitric oxide-induced lipid peroxidation and death of retinal photoreceptors: A comparison with other anti-glaucoma drugs. Invest Ophthalmol Vis Sci.

[6] Masini E, Cuzzocrea S, Mazzon E, Marzocca C, Mannaioni PF, Salvemini D (2002). Protective effects of M40403, a selective superoxide dismutase mimetic, in myocardial ischaemia and reperfusion injury *in vivo*. Br J Pharmacol.

[7] Bressler NM, Arnold J, Benchaboune M, Blumenkranz MS, Fish GE, Gragoudas ES (2002). Verteporfin therapy of subfoveal choroidal neovascularization in patients with age-related macular degeneration: Additional information regarding baseline lesion composition’s impact on vision outcomes-TAP report No.3. Arch Ophthalmol.

[8] Cortas NK, Wakid NW (1990). Determination of inorganic nitrate in serum and urine by a kinetic cadmium-reduction method. Clin Chem.

[9] Ohkawa H, Ohishi N, Yagi K (1979). Assay for lipid peroxides in animal tissues by thiobarbituric acid reaction. Anal Biochem.

[10] Lowry OH, Rosebrough NJ, Farr AL, Randall RJ (1951). Protein measurement with the Folin phenol reagent. J Biol Chem.

[11] Zacks DN, Ezra E, Terada Y, Michaud N, Connolly E, Gragoudas ES (2002). Verteporfin photodynamic therapy in the rat model of choroidal neovascularization: Angiographic and histologic characterization. Invest Ophthalmol Vis Sci.

[12] Paskowitz DM, Nune G, Yasumura D, Yang H, Bhisitkul RB, Sharma S (2004). BDNF reduces the retinal toxicity of verteporfin photodynamic therapy. Invest Ophthalmol Vis Sci.

[13] Reinke MH, Canakis C, Husain D, Michaud N, Flotte TJ, Gragoudas ES (1999). Verteporfin photodynamic therapy retreatment of normal retina and choroid in the cynomolgus monkey. Ophthalmology.

[14] Murphy MP (1999). Nitric oxide and cell death. Biochim Biophys Acta.

[15] Yamamoto F, Ohgari Y, Yamaki N, Kitajima S, Shimokawa O, Matsui H (2007). The role of nitric oxide in delta-aminolevulinic acid (ALA)-induced photosensitivity of cancerous cells. Biochem Biophys Res Commun.

[16] Ali SM, Olivo M (2003). Nitric oxide mediated photo-induced cell death in human malignant cells. Int J Oncol.

[17] Gupta S, Ahmad N, Mukhtar H (1998). Involvement of nitric oxide during phthalocyanine (Pc4) photodynamic therapy-mediated apoptosis. Cancer Res.

[18] She H, Nakazawa T, Matsubara A, Hisatomi T, Young TA, Michaud N (2007). Reduced photoreceptor damage after photodynamic therapy through blockade of nitric oxide synthase in a model of choroidal neovascularization. Invest Ophthalmol Vis Sci.

[19] Saczko J, Kulbacka J, Chwiłkowska A, Lugowski M, Banaś T (2004). Levels of lipid peroxidation in A549 cells after PDT *in vitro*. Rocz Akad Med Bialymst.

[20] Du HY, Olivo M, Tan BK, Bay BH (2003). Hypericin-mediated photodynamic therapy induces lipid peroxidation and necrosis in nasopharyngeal cancer. Int J Oncol.

[21] De La Paz MA, Zhang J, Fridovich I (1996). Antioxidant enzymes of the human retina: Effect of age on enzyme activity of macula and periphery. Curr Eye Res.

